# Rare Presentation of a Primary Cardiac Mass: Insights Into Multimodal Imaging, Histopathology, and Surgical Management

**DOI:** 10.1016/j.jaccas.2025.103931

**Published:** 2025-07-09

**Authors:** Ashraf Samhan, Sowda Ahmed, Alana A. Lewis, Paul C. Cremer, Lamis ElHarake, Jon W. Lomasney, Douglas R. Johnston, Mohamed Al-Kazaz

**Affiliations:** a Feinberg School of Medicine, Northwestern University, Chicago, Illinois, USA; b Division of Cardiology, Bluhm Cardiovascular Institute, Chicago, Illinois, USA; c Division of Radiology, Northwestern Memorial Hospital, Chicago, Illinois, USA; d Division of Pathology, Northwestern Memorial Hospital, Chicago, Illinois, USA; e Division of Pharmacology, Northwestern Memorial Hospital, Chicago, Illinois, USA; f Division of Cardiac Surgery, Bluhm Cardiovascular Institute, Chicago, Illinois, USA

**Keywords:** computed tomography, imaging, magnetic resonance sequences, pericardial effusion, positron emission tomography, right ventricle, tamponade, thoracotomy

## Abstract

Primary cardiac tumors are exceedingly rare, with cardiac hemangiomas constituting 1% to 3% of benign cardiac tumors, making diagnosis and management challenging. A 49-year-old female presented with progressive chest tightness over several years. Multimodal imaging revealed a large cardiac mass within the pericardial space, encasing the left anterior descending artery. Following an inconclusive tissue sampling attempt, surgical resection was performed to guide therapy. Histopathology confirmed cardiac cavernous hemangioma. Multimodal imaging is crucial for characterizing cardiac masses and guiding biopsy and treatment strategies (eg, surgical for sarcoma if feasible versus nonsurgical for lymphoma). Cardiac hemangiomas often present with nonspecific symptoms. Surgical resection remains the definitive treatment, providing symptom relief and histopathologic confirmation.

## Graphical Abstract

**Figure:**
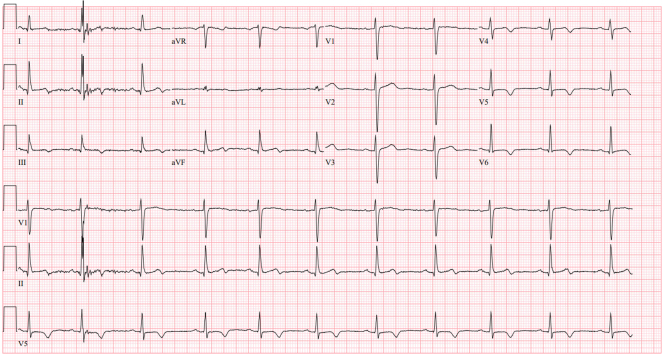

## History of Presentation

A 49-year-old White female presented to her primary care provider for intermittent, non-exertional supine chest tightness that had been increasing in frequency over the past several years. She denied any constitutional symptoms and remained physically active without functional limitations. Her physical examination was unremarkable. Initial vital signs were notable for a blood pressure of 118/82 mm Hg, heart rate of 73 beats/min, and oxygen saturation of 98% on room air.

**Take-Home Messages**

• Primary cardiac tumors should be considered in the differential diagnosis for atypical chest discomfort.
• Accurate characterization of cardiac masses requires a combination of imaging modalities (echocardiography, positron emission tomography, computed tomography, and magnetic resonance imaging) and tissue biopsy to guide diagnosis and treatment planning.
• For certain malignant tumors (eg, sarcomas) and symptomatic benign cardiac tumors, surgical resection is the treatment of choice.
• Other malignant neoplasms such as lymphoma are managed nonoperatively; therefore, tissue sampling and confirming the histopathologic diagnosis are of critical importance.

## Past medical history

The patient’s medical history was significant for basal cell carcinoma status post resection, prior bariatric surgery, gastroesophageal reflux disease, and perimenopause. She also has a family history of premature coronary artery disease (CAD), with her mother undergoing multivessel coronary artery bypass grafting at 55 years of age. There is no family history of malignancy.

## Differential diagnosis

The differential diagnosis included esophagitis, esophageal spasm, costochondritis, muscular strain, pericarditis, pleuritis, myocardial ischemia, Prinzmetal angina, hiatal hernia, and pulmonary embolism.

## Investigations

**Figure 1:**
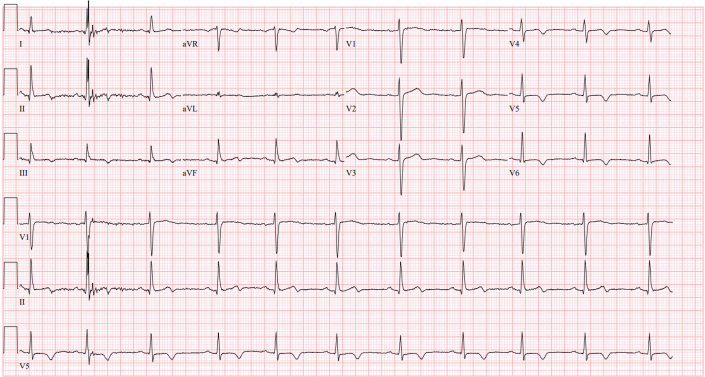
Electrocardiogram Normal sinus rhythm is shown with low voltage in the precordial leads and T-wave inversions anterolaterally.

Her complete blood count, comprehensive metabolic panel, C-reactive protein, sedimentation rate, and thyroid-stimulating hormone were within normal limits. Laboratory findings were notable for an elevated D-dimer of 1,371 ng/mL and high-sensitivity troponin of 3 pg/μL. QuantiFERON TB Gold and urine human chorionic gonadotropin were negative. The initial electrocardiogram showed normal sinus rhythm with low voltage in the precordial leads and anterolateral T-wave inversions (Figure 1).

**Figure 2:**
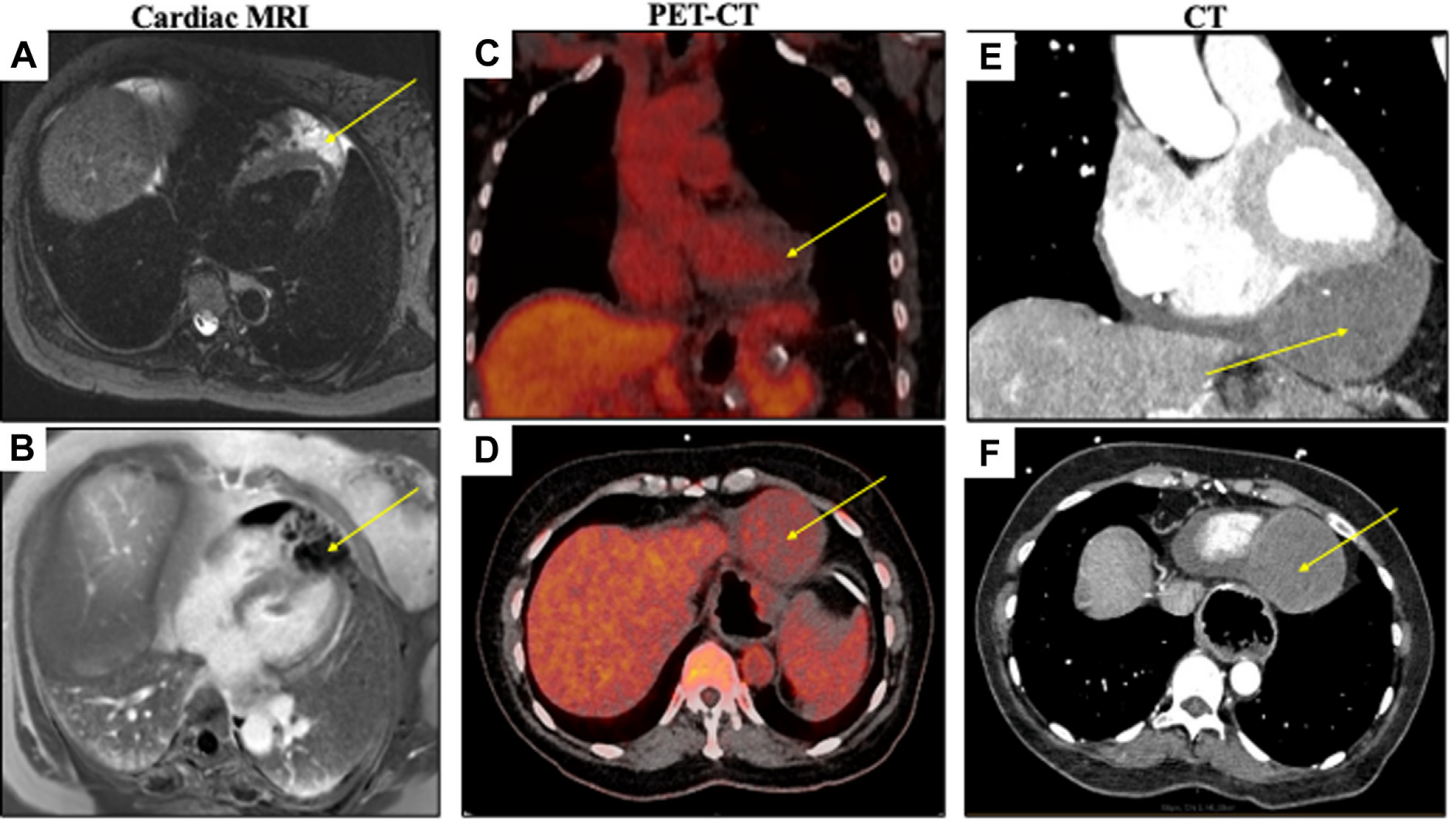
Multimodality Imaging Cardiac magnetic resonance, positron emission tomography (PET), and computed tomography (CT) are shown. (A) Cardiac MRI with T2 signaling and (B) late gadolinium enhancement showing a 7.7 × 5.7 cm well-circumscribed, multiseptated, heterogeneously enhancing mass in the pericardial space, inferior to the left ventricular apex. (C, D) PET-CT showing a 6.0 × 6.4 × 7.8 cm solid mass arising from the left pericardial space demonstrating low-level radiotracer uptake. (E, F) Coronal and axial CT images show a 7.0 × 6.9 × 4.2 cm intrapericardial mass abutting the inferior left ventricular wall and apex and encasing the distal left anterior descending artery.

Given the elevated D-dimer, she was referred for an urgent computed tomography (CT) angiogram with pulmonary embolism protocol, which revealed an 8-cm solid mass likely arising from the pericardium with mass effect on the inferior aspect of the heart. An acute pulmonary embolism and aortic dissection were not identified. These findings prompted admission for an expedited workup, during which further diagnostic imaging was performed. A CT angiogram revealed no evidence of CAD but did show a cardiac mass in the pericardial space with concern for local myocardial invasion at the apical level and encasing the distal left anterior descending (LAD) artery (Figure 2). Transthoracic echocardiography (TTE) showed a heterogeneous mass in the pericardial space close to the apical and inferior portions of the heart, along with a small pericardial effusion (Video 1). Cardiac magnetic resonance (CMR) revealed a well-circumscribed, multiseptated, heterogeneously enhancing 7.7 × 5.7 cm mass inferior to the ventricles with local invasion with enhancement of the septae within this mass (Figure 2). The mass showed a high fluid component, as indicated by the high T2 signal, suggesting a fluid component or necrosis. Finally, a cardiac positron emission tomography–CT was performed to evaluate for malignancy, which confirmed a 6.0 × 6.4 × 7.8 cm solid mass arising in the left pericardial space with low-level radiotracer uptake and deemed indeterminate (Figure 2). It showed no other areas of pathologic uptake. There was no evidence of hypermetabolic lymph node enlargement.

## Management

Multidisciplinary discussions were held to assess the feasibility of performing a biopsy of the mass to rule out malignancy, such as a rapidly growing cardiac lymphoma with central necrosis, which can have a similar appearance. This was considered as an alternative to direct surgical radical resection, which is typically pursued in sarcoma cases when appropriate. Given the location of the mass and its proximity to adjacent structures, interventional radiology deemed ultrasound- or CT-guided biopsy too high-risk.

The patient underwent a transesophageal-guided core needle biopsy with cardiac surgery; however, the sample results were inconclusive. One day later, she developed chest pain and hypotension and was found to have an enlarging pericardial effusion with tamponade physiology. The decision was made to sample the mass and evacuate the pericardial effusion via a minithoracotomy and pericardial window. The sample was then sent for frozen section, and intraoperative pathologic examination revealed patchy lymphocytic infiltrate and focal atypical cells, but no lymphoma cells were identified.

**Figure 3:**
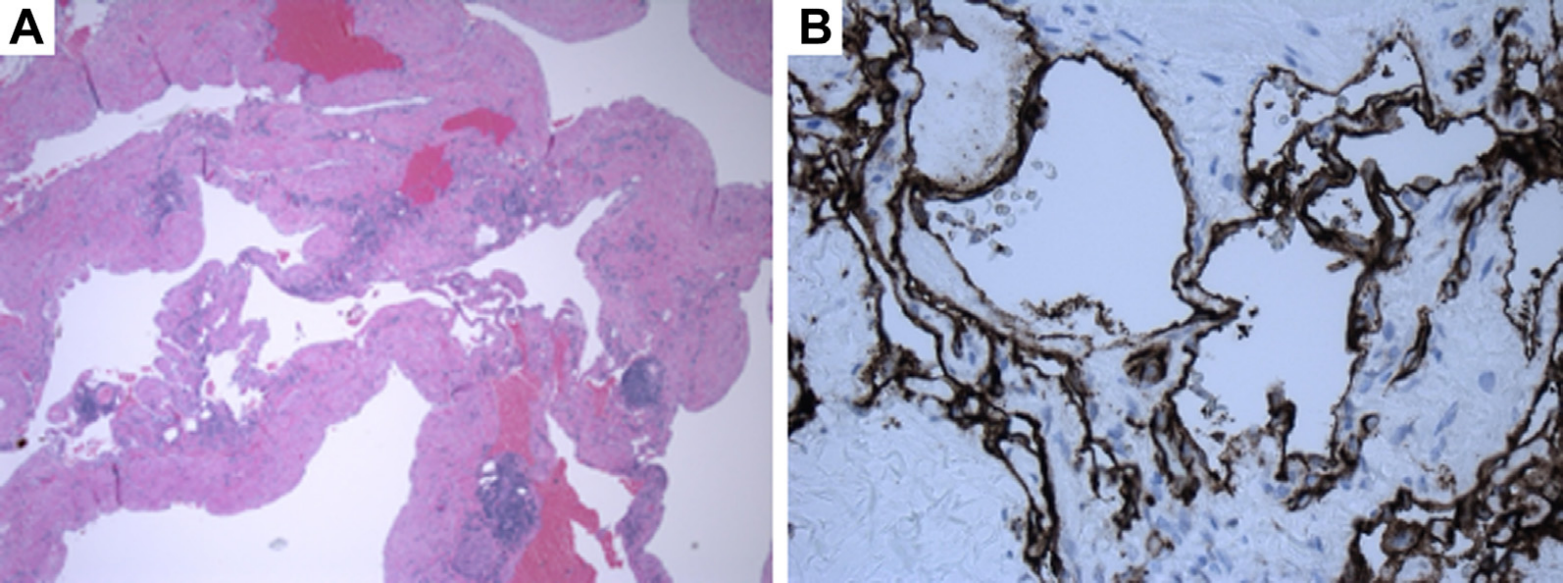
Histology Histological image of the cavernous hemangioma obtained from surgical resection and stained with hematoxylin and eosin. (A) Large, blood-filled vascular spaces lined by a single layer of flat endothelium are confirmed by (B) CD31 staining and supported by fibrous septa.

Subsequently, the patient underwent a full sternotomy, during which the mass was observed to arise from the apical myocardium without any evident areas of invasion. It was also found to encase the distal LAD without causing significant stenosis. She underwent complete mass resection, ligation of the distal LAD, and bovine pericardial patch repair of the right ventricle. Her postoperative course was complicated by atrial fibrillation with rapid ventricular rate that resolved with amiodarone and metoprolol. The final histopathologic examination revealed a benign vascular neoplasm consistent with a cavernous hemangioma (Figure 3). She was discharged home 1 week after mass resection.

## Outcome and follow-up

She was seen in primary care, cardiology, and cardiac surgery clinics within 1 month of her mass resection, where she reported resolution of her initial symptoms. She is also participating in cardiac rehabilitation, and a repeat CT scan showed no recurrence of the pericardial mass or effusion.

## Discussion

Primary cardiac tumors are rare, with an incidence of approximately 0.002% to 0.03% in autopsy studies.^1^ The differential diagnosis for a cardiac mass includes benign and malignant conditions (Table 1). Benign lesions often include pericardial cysts and lipomas, whereas malignant masses may include mesothelioma, sarcomas, lymphoma, and metastatic tumors. Cardiac hemangiomas, benign vascular tumors, account for only 1% to 3% of all benign cardiac tumors. Histologically, they are subclassified as cavernous, capillary, or arteriovenous. Despite their rarity, they pose a clinical challenge due to their indolent course and often nonspecific, variable symptoms. Symptoms are frequently mild or overlooked, leading to delayed diagnosis for years. This case highlights a rare presentation of a cardiac cavernous hemangioma, and the diagnostic insights achieved through multimodal imaging and surgical intervention.

**Table 1:** Differential Diagnosis of Cardiac Masses in the Pericardial Space and Diagnostic Approach Based on Multimodal Imaging

Condition	Key Features	Key Imaging Characteristics	Histologic Features
Benign lesions			
Pericardial cysts	Well-defined, fluid-filled structure	CT: Thin-walled, non-enhancing lesion MRI: Hyperintense on T2-weighted images, no gadolinium enhancement	Single layer of cuboidal or flat mesothelial cells
Lipomas	Encapsulated fatty mass	CT: Fat attenuation MRI: Bright on T1-weighted, suppressed on fat-saturation sequences	Well-encapsulated, mature fat cells
Cavernous hemangiomas	Rare vascular tumors	CT: enhancing soft tissue mass MRI: high vascularity with intense gadolinium enhancement	Large, dilated vascular spaces lined by a single layer of endothelial cells and filled with blood
Malignant lesions			
Mesothelioma	Rare; associated with asbestos exposure	CT: nodular, thickened pericardium MRI: irregular pericardial enhancement, often diffuse	Atypical mesothelial cells forming papillary or tubular structures; immunoreactive for markers including cytokeratin, calretinin, CA125
Sarcoma	Rapidly growing mass; may cause obstruction or pericardial effusion	PET-CT: high FDG uptake in primary and metastatic lesions MRI: heterogeneous signal intensity with gadolinium enhancement	Pleomorphic spindle or round cells with marked nuclear atypia and areas of necrosis
Lymphoma	Often linked to immunocompromise; rapid pericardial effusion	CT: bulky mass adjacent to pericardium PET-CT: increased FDG uptake MRI: T2 hyperintensity, moderate enhancement	Diffuse proliferation of large, atypical lymphoid cells with high nuclear-to-cytoplasmic ratios, prominent nucleoli, and positive immunohistochemical staining for B-cell markers
Metastatic tumors	Secondary to lung, breast, or melanoma; symptoms of pericardial effusion	CT/MRI: irregular masses with enhancement PET-CT: FDG-avid lesions in pericardium	Infiltrating malignant cells resembling the primary tumor

CT = computed tomography; FDG = fludeoxyglucose; MRI = magnetic resonance imaging; PET = positron emission tomography.

Cardiac cavernous hemangiomas comprise large, dilated blood vessels and are typically classified as benign vascular neoplasms. These tumors consist of a network of blood vessels and can exhibit various histopathologic features, including patchy lymphocytic infiltrates, necrosis, and occasional focal atypical cells. Cavernous hemangiomas are characterized histologically by well-formed vascular spaces lined by endothelial cells, which may contain areas of thrombosis or hemorrhage.^2^ Anatomically, they can involve any part of the heart, but most reported cases have involved the right atrium and ventricle.^3^ Although these tumors are benign, their size and location can lead to significant clinical complications, such as pericardial effusions causing tamponade or compression of cardiac structures leading to myocardial ischemia, outflow obstruction, and arrhythmias.^2^^,^^4^^,^^5^

Advanced imaging plays a critical role in the diagnosis and surgical planning of cardiac tumors (Table 1). CT and CMR imaging are particularly valuable in providing detailed anatomical and tissue characterization. CMR is especially useful for identifying high signal intensity on T2-weighted images and assessing the effect of the tumor on cardiac function and surrounding structures. Patel et al^6^ compared the diagnostic performance of CMR and TTE in 50 patients with histologically confirmed cardiac or pericardial masses. Their findings demonstrated that CMR was superior in identifying pericardial masses and provided significantly more accurate histopathologic diagnoses.^6^ Cardiac CT is also helpful for evaluating the extent of involvement with adjacent structures; in our case, CT angiogram ruled out CAD while visualizing an intrapericardial mass encasing the LAD.

Although multimodal imaging is invaluable for the differential diagnosis of cardiac tumors, histopathology remains essential for guiding treatment planning. Current literature describes 3 methods for obtaining tissue samples from cardiac masses, each with its risks and benefits: percutaneous ultrasound-guided core needle biopsy, CT-guided fine-needle aspiration, and open biopsy via thoracotomy or sternotomy.^7^^,^^8^ In cases such as this, multidisciplinary discussions with interventional radiology and cardiac surgery are crucial to determining the safest approach, especially given the elevated risk of post-biopsy hemorrhagic pericardial effusion associated with hemangiomas. Because of initial concerns that the patient’s mass was malignant based on imaging, proceeding with a minimally invasive approach was preferred rather than proceeding directly to sternotomy.

The treatment of a cardiac mass depends on its underlying etiology, location, and effect on the myocardium and pericardium. For malignant lesions such as lymphoma, treatment typically involves a combination of chemotherapy, immunotherapy, radiation, and/or surgical resection.^9^ Surgical resection is the primary treatment for symptomatic or large cardiac cavernous hemangiomas.^2^^,^^3^^,^^10^ This approach is often necessary to alleviate symptoms, prevent complications, and achieve a definitive histologic diagnosis. Although complete resection is preferred, debulking may be performed if full excision is not feasible because of the location of the tumor or involvement with critical structures.^10^ Following complete resection, the prognosis is generally favorable with a low recurrence rate.^2^

## Conclusions

Cardiac cavernous hemangiomas are rare, benign vascular tumors that are typically asymptomatic but can present with nonspecific symptoms depending on their size and location. Multimodal imaging is essential for accurate diagnosis and for guiding surgical management. For symptomatic cardiac tumors, surgical resection remains the definitive treatment, offering both symptom resolution and histopathologic confirmation. With timely intervention, the prognosis for patients with cardiac cavernous hemangiomas is generally favorable, as shown by the successful outcome in this case.

## Funding Support and Author Disclosures

Dr Al-Kazaz has received research grants and speaking Honoria from Kiniksa Pharmaceuticals; and has received consulting fees from Edwards LifeSciences. All other authors have reported that they have no relationships relevant to the contents of this paper to disclose.

## Appendix

Video 1 Transthoracic Echocardiogram Transthoracic echocardiogram showing a 4.36 × 5.90 cm heterogeneous mass in the apical and inferior portion of the heart adjacent to the ventricular region of the pericardium with a moderate to large pericardial effusion.
